# Examination of public perceptions of microbes and microbiomes in the United States reveals insights for science communication

**DOI:** 10.1371/journal.pone.0312427

**Published:** 2024-10-21

**Authors:** Katherine Kokkinias, Katherine Pruneski, Kelly Wrighton, Nicole Kelp

**Affiliations:** 1 Department of Microbiology, Immunology and Pathology, Colorado State University, Fort Collins, Colorado, United States of America; 2 Department of Soil and Crop Sciences, Colorado State University, Fort Collins, Colorado, United States of America; Swiss Paraplegic Research, SWITZERLAND

## Abstract

Within a changing research and media landscape, misconceptions and misinformation about microorganisms and microbiomes can arise, necessitating improvements in science communication practices through insights in public perceptions of the microbial world. Yet, little is known about public perceptions of microorganisms and microbiomes, making it difficult to develop tailored messaging. Here we perform an inductive thematic analysis with interviews and surveys from thirty adults across the United States to identify key factors to enhance microbial science communication efforts. Together, our results underscore the importance of 1) recognizing the existing and desired future knowledge of an audience, 2) aligning with broader socio-scientific issues that resonate with people in relevant channels using social networks, 3) fostering collaboration between microbiologists, social scientists, and communicators to improve messaging, and 4) appealing to people’s values and emotions to establish meaningful connections. This study concludes that non-microbial interests, such as an interest in health and wellness, may lead to acquisition of microbial knowledge and that people want scientists to share microbial messages preferably on platforms like social media. Additionally, we identified confusion about microbial terms and a desire to understand human-centric benefits of microorganisms and microbiomes. We suggest that microbiologists partner with science communicators to develop microbial messaging, capitalizing on connections to non-microbial interests and appealing to people’s microbial worldview.

## Introduction

Microorganisms (microbes) and microbiomes are vital to life on earth and drive processes related to carbon and nutrient cycling, produce secondary compounds like antibiotics, and facilitate gas exchange which have ramifications on human, animal, plant, and soil health [1–3]. Despite their broad impacts, the term "microbes" is vague and could refer to bacteria, viruses, fungi, or protozoa [4]. The term “microbiome” describes microbes from an ecological perspective and refers to a community of microorganisms and their genomes in an environment at a particular point in time, including both biotic and abiotic factors [5]. The importance of science literacy of microbial terms and functions becomes more apparent with the increase of emerging infectious diseases and global environmental challenges related to climate change [6–9]. During the COVID-19 pandemic, the complex, ever-changing landscape of microbial research made science communication efforts challenging, leading to misinformation and an “infodemic”, where there was an overwhelming amount potentially false or misleading information on the subject [10–13]. With the abundance and availability of information, discerning what is factual and false can be difficult.

At the same time, advances in science pave the possibility of new therapeutics through mechanistic discoveries about microbes and microbiomes [14, 15]. To highlight these discoveries, there has been a global increase in media coverage and the growth of a multi-billion dollar pre- and probiotic industry based on microbiome research which often overstate the efficacy or importance of research findings [16–18]. With increased opportunity for awareness of microbial functions and their impacts, understanding public perceptions of microbes and microbiomes to guide science communication efforts is essential to public health and scientific advocacy.

Microbes are often associated with negative outcomes such as disease, even though much less than 1% of microbes are considered pathogenic [19]. This association with disease can lead to uncertainty and negative emotions, like fear or anxiety [6, 20–22]. Importantly, negative emotions can influence thoughts, learning, and decision-making [23–26]. These perceptions can influence health behaviors which can lead to the rejection of therapeutic interventions, increase health disparities, or in some cases, result in loss of life [10, 27, 28]. Researchers in the fields of science education have investigated school-aged children’s perceptions of microbes and found that students associate microbes with something harmful [22]. Additionally, studies have examined public perceptions of specific infectious diseases such as COVID-19, Mpox, West Nile virus, or malaria, finding misconceptions about causes and prevention strategies as well as emotions such as fear and apathy [29–32]. Together, misconceptions about microbes can delay lifesaving preventative care and can result in hesitance towards public health recommendations [6, 10, 33–37]. Similarly, perceived risk research on fecal microbiome transplants found feelings of disgust stimulated a greater perceived risk of microbiome research and therapies [20]. While this body of research examines perceptions of disease-causing agents or therapeutics, little is known about public perceptions of microbes and microbiomes in general. Furthermore, few studies have asked for stakeholder input into future microbial science communication.

Knowledge about the audience is essential for any communication campaign but is especially important with complex subjects that are characterized by uncertainty or elicit emotions like fear [10, 25, 26, 38–41]. Different audiences have different perceptions of such issues, developed through personal experiences and beliefs which can influence health behaviors and actions [30, 35–37, 42]. To create tailored messaging to meet science communication goals, there needs to be an understanding of public perceptions. For example, research in public perceptions of climate change has identified six worldviews towards climate change amongst Americans: alarmed, concerned, cautious, disengaged, doubtful and dismissive [43, 44]. Climate change communication research has focused on targeting messaging to these groups to evoke change [44–47]. Such audience analysis regarding microbial perceptions is lacking.

Multiple theories examine how people respond to infectious disease and elements to consider in communication research. Briefly, the Theory of Motivated Information Management provides a framework for analyzing how individuals manage emotions and information in the context of uncertainty; additionally, it focuses on how people assess the outcomes of their information-seeking and the efficacy of the communication process they undertake to gain that information [41]. Correspondingly, Lasswell’s model of communication [48, 49] frames communication into five factors: 1) Who is the communicator? 2) What is the message? 3) What channel or medium is it being shared through? 4) Who is the audience? 5) What is the effect? Using these theoretical frameworks, our goal was to identify previous knowledge, sources of information, desired messages, and suggestions for future science communication efforts about microbes and microbiomes. Specifically, we utilize our data and the existing theoretical frameworks to address the following research questions:

RQ1: What do people know about microbes and microbiomes?
RQ2: Where and why have they learned this information?
RQ3: What do people want to know about microbes and microbiomes?
RQ4: How do people want that information shared and who do they think should share that information?
RQ5: How do emotions impact public perceptions about microbes and microbiomes?

Through interviews and a survey, participants shared their experiences and preferences for future microbial science communication campaigns. We assessed where people have learned about microbes and microbiomes, what microbial information people want, how to share it, who should share it, and how other interests and emotions might affect the reception of that information. Together, these results can be used to create more effective, relevant science communication about microbes and microbiomes.

## Methods

### Participant recruitment and interviews

In the summer of 2023, adults across the United States were recruited via flyers and social media to participate in semi-structured interviews about microbes and microbiomes. Recruitment occurred from March 11, 2023 through August 21, 2023. Thirty participants were selected to be interviewed on a first-come first-served basis. The interview facilitation guide was developed to stimulate conversation around microbes and microbiomes. Questions pertained to definitions of microbes and microbiomes, their functions, personal and peer perceptions of these topics and science communication. Interviews were chosen for the flexibility of assessing participants’ knowledge, allowing researchers to probe explanations and clarify any inconsistencies. As interviews proceeded, the interview facilitation guide was revised to include follow-up questions commonly mentioned by the participants. A final version of the interview facilitation guide is available in the **S1 File**.

**Fig 1:**
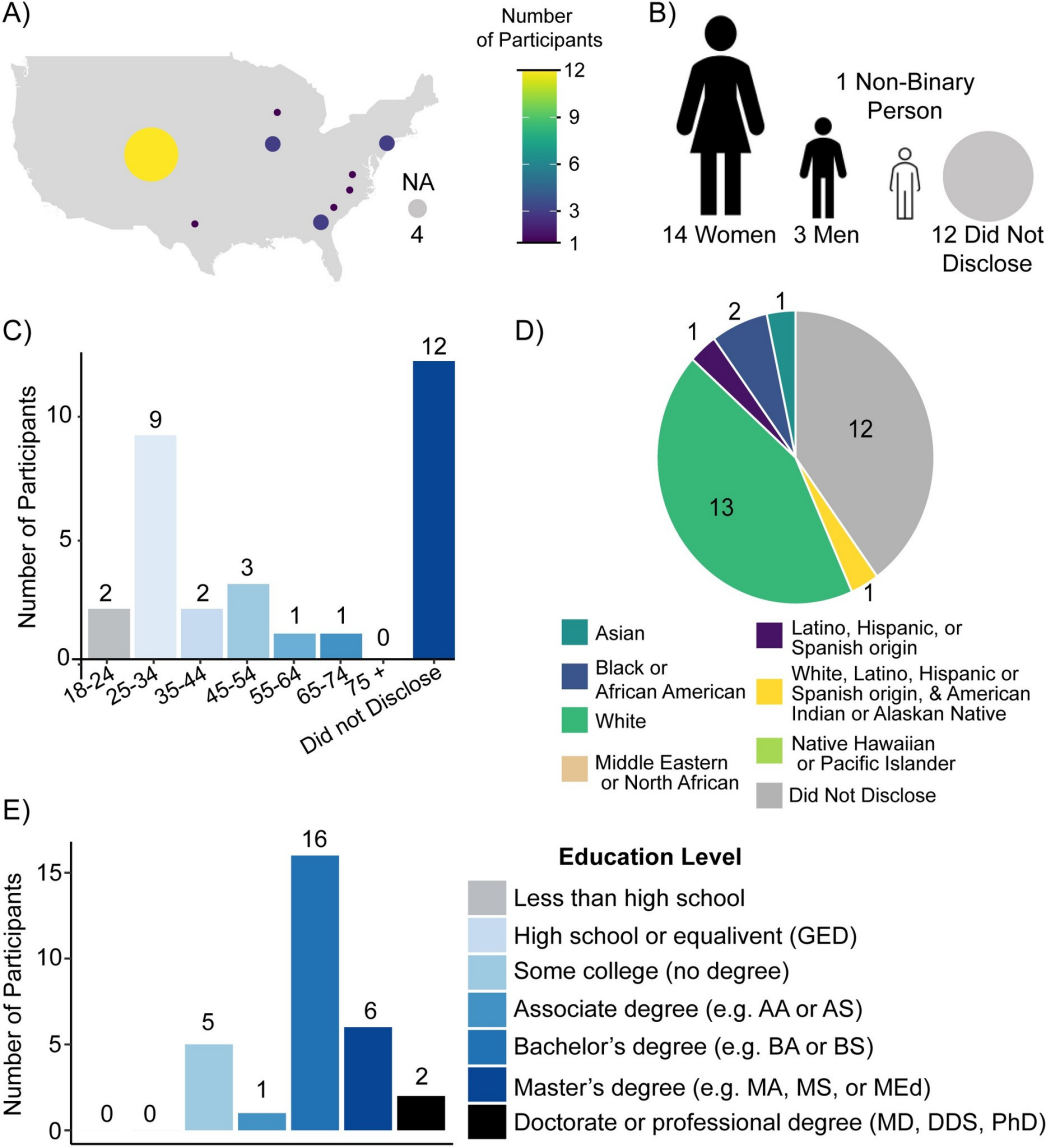
Participant demographics. Demographics of participants (n = 30) are depicted including A) where participants live in the United States, B) participants’ gender (women, men, or non-binary), C) age range of participants, D) participants’ ethnicity, and E) participants’ education level. Demographics information provided during interviews was included for participants who did not complete the survey.

These interviews were conducted either in-person or via Zoom based on the preference and physical location of the participant. This study was approved by the Institutional Review Board at Colorado State University (IRB: #4304). All interviews were recorded with the participants’ approval. Participants provided verbal consent for the study during the interview, which was recorded via the transcript. Interviews were on average ~27 minutes and a survey with demographics questions as well as a question regarding science curiosity was sent out via email after the interview. The science curiosity survey question was developed based on a previously published science curiosity scale [50, 51]. Surveys were sent via email and collected via Qualtrics. For the demographics and science curiosity survey via Qualtrics, participants provided written consent. A document containing the questions listed in the survey is provided in the **S2 File**. The survey had a 60% (18/30) response rate. Our participants were predominately white (43%), between 25 and 34 years of age (30%), and all had received at least a high school diploma or equivalent (**Fig 1**). When demographics information was provided during the interview, it was included in Fig 1 for participants who did not complete the survey. Participants were compensated for their time with a $25 gift card. Participants’ transcripts were deidentified and transcribed before analysis.

### Interview analysis

After transcription of the recorded interviews, we performed inductive coding reliability thematic analysis. Coding reliability thematic analysis allows for some quantification of themes and sub-themes to identify trends in the data [52, 53]. Two researchers (KK and KP) reviewed five interviews to identify recurring themes and trends in the data. After the two researchers met to discuss their notes, they collapsed similar themes to create an initial codebook. The constant comparison method was used to compare quotes to themes, modify descriptions and examples, and determine if additional themes were necessary [54]. Together, these researchers revised the codebook until they were confident it captured common themes in the data and no new themes emerged. A final copy of the codebook can be found in **Table 1**. To determine intercoder reliability, 10% of interviews (that is, three interviews) were independently coded using the codebook, and codes were compared within each line as defined by MAXQDA software (v 22.8.0) [55]. Cohen’s κ intercoder score was acceptable (κ = 0.783) as was percent agreement (95%) [56]. As with other studies [57–60], intercoder reliability metrics were used to promote rigor and reflexivity in our data by identifying nuance in how the two researchers were coding the data which was discussed among the researchers. The remaining interviews were divided among the two researchers (KK and KP) for coding using the MAXQDA software (v 22.8.0) [55]. Through codebook thematic analysis of the data, a frequency table of the sub-theme examples was generated by KK and KP to visualize the proportion of participants that mentioned a sub-theme example (**Table 2)**. Sub-theme examples are listed if at least two participants mentioned that example. Within the interviews, some participants (n = 19 participants) mentioned interests that led to microbial knowledge, while other participants did not mention interests that led to microbial knowledge (n = 11 participants). This classification was based on the interview transcript.

**Table 1:** Codebook. Codebook including themes, sub-themes, descriptions of sub-themes, and example quotes from participants generated through inductive thematic analysis.

Themes	Sub-themes	Description	Example Quotes
Previous Knowledge	Microbes Features and Functions	A physiological feature or function of microbes	“It’s something microscopic, so it’s something that you can’t see with the naked eye. It’s a small life form. Affects our daily lives and you. . . they are necessary. That can sometimes be bad.” (P11)
	Microbiome Features and Functions	A physiological feature or function of microbiomes	“Because there are lots of bacteria that grow in the soil and break down different nutrients and breakdown some of the fibrous materials and leftover parts that are in soil. So, like when we till our gardens turns over the bacteria as well. That’s in there and helps break it up.” (P8)
Topics of interest that led to microbial knowledge	Nature	An interest in nature or the environment	“One thing that that comes to mind is… I’m pretty passionate about climate change, and I have found some of the research at a high level very interesting… [I am interested] in a microbe that could eat away at plastic. . .” (P3)
	Food systems	An interest in food systems (i.e., gardening, farming, agriculture)	“I’m very conscious of the environment. I’m very conscious of my family, with what they eat. I like to garden, I’m a beekeeper.” (P25)
	Health and Wellness	An interest in health and wellness including (i.e., exercise, nutrition, gut health, holistic or natural remedies)	‘I’m just a person that is interested in fitness and nutrition. You know, there’s a lot of “gut biome” stuff that comes up.” (P15)
Information seeking processes (past and desired future)	Communicator	A person, professional, educational institution, or governmental institution who has or should communicate about microbes/microbiomes	“Yeah, my brother went on a real kick about it [the gut microbiome]. So, he lectured me about it for a while. This was probably five or six years ago. So, I got some information from him.” (P4)
	Channel or Medium	A channel or medium where participants have received microbial information or think should be used to share information in the future	“Where I’m at in life now. . . . Probably just see it on Instagram or a cool YouTube video. I think that’s where I do most of my learning. It’s either a friend recommends me a book, or I just am on YouTube at like 11:30 at night and I’m just clicking whatever sounds interesting.” (P12)
	Communication techniques	A communication technique that should be used to communicate information about microbes/microbiomes	“I’m more of like a visual learner, so I would like some sort of video that kind of shows what’s going on, how they interact with everything. It’d be kind of hard, since they’re so small, but you know, diagrams help.” (P17)
	Desired messages	A topic about microbes and microbiomes that participants would like to know or think others should know	“I think we should demonstrate the significance of them and show why they, why they’re necessary for us. And how they help us. How they help us humans and our just our daily lives.” (P10)
Emotions affecting microbial worldview	Emotions towards self	A participants’ emotions about themselves related to microbes/microbiomes	I want to find out more about it [microbes and microbiomes]. I’m interested now. I feel like going to the library tomorrow and just like read up more about it… and seek more information… So maybe ask my doctor for information about that. (P29)
	Perceptions of others	Characteristics ascribed towards others by the participant related to microbes/microbiomes	“I feel like the general perception from people who don’t have a STEM background is that like microbes are bad. Like, you have to disinfect everything because you don’t want the microbes. People that have the STEM backgrounds… are very excited about microbes and all of the potential implications that they have for understanding certain processes in the world.” (P1)

**Table 2:** Frequency of sub-theme examples. The table lists the percent of participants (n = 30) who mentioned the listed example of each sub-theme. Sub-theme examples are listed from greatest to least frequency of mention within each sub-theme. Sub-theme examples are included if at least two participants mentioned that example.

Themes	Sub-themes	Sub-themes examples	% of participants mentioning this example (n = 30)
Previous knowledge	Microbes Features and Functions	a. Neutral Feature or Function	96.7% (29)
		b. Positive Feature or Function	93.3% (28)
		c. Negative Feature or Function	70% (21)
	Microbiomes Features and Functions	a. Neutral Feature or Function	100% (30)
		b. Positive Feature or Function	93.3% (28)
		c. Negative Feature or Function	53.3% (16)
Topics of interest that led to microbial knowledge	Nature	a. Climate Change	16.7% (5)
	Food systems	a. Gardening and Composting	16.7% (5)
		b. Farming/ Agriculture	13.3% (4)
	Health and Wellness	a. Nutrition and gut health	36.7% (11)
		b. Holistic & natural medicine	30% (9)
		c. Exercise and fitness	6.7% (2)
Information seeking processes (past and desired future)	Communicator	a. Profession (i.e. doctors, scientists, etc.)	56.7% (17)
		b. Individual	43.3% (13)
		c. Educational Institutions (i.e. museums, universities)	40% (12)
		d. Governmental agencies (i.e. NIH, CDC)	23.3% (7)
	Channel/ Medium	a. Class	80% (24)
		b. Social media	56.7% (17)
		c. Internet	46.7% (14)
		d. Word of mouth	43.3% (13)
		e. News	43.3% (13)
		f. Source Amnesia	36.7% (11)
		g. Advertisements	30% (9)
		h. Books	26.7% (8)
		i. Podcast	26.7% (8)
		j. TV/Movies	20% (6)
		k. Research Articles	13.3% (4)
	Communication techniques	a. Visual format (i.e. video, diagrams)	63.3% (19)
		b. Trusted sources	46.7% (14)
		c. Plain language	43.3% (13)
		d. Short	36.7% (11)
		e. Connect to interests	33.3% (10)
		f. Partner with communicators	30% (9)
		g. Shared identity	16.7% (5)
		h. Humor	6.7% (2)
	Desired messages	a. General microbial knowledge (i.e. definitions)	76.7% (23)
		b. Disease avoidance and Health Promotion	53.3% (16)
		c. Microbial Benefits to humans	53.3% (16)
		d. Ecological roles of microbes	46.7% (14)
		e. Microbial Benefits to the environment	30% (9)
		f. Microbial Benefits to industry	23.3% (7)
Emotions affecting microbial worldview	Emotions towards self	a. Lack of Self-efficacy	96.7% (29)
		b. Apathy	56.7% (17)
		c. Self-efficacy	43.3% (13)
		d. Curiosity	43.3% (13)
		e. Cognitive overload	26.7% (8)
		f. Fear	23.3% (7)
	Perceptions of Others	a. Ignorant	90% (27)
		b. STEM Expertise	70% (21)
		c. Knowledgeable	66.7% (20)
		d. Disease-focused	63.3% (19)
		e. Apathy	50% (15)
		f. Intimidation	36.7% (11)

### Figures and statistics

Figures and statistics were generated using Excel or R (ggplot2 package v3.3.5, dplyr package v1.1.0, readxl package v1.4.2, and viridis package v0.6.2) [55–58] and modified using Adobe Illustrator (v27.6.1). All available scripts for generating the figures can be found here (https://github.com/Kokkinias/Public-Perceptions-of-Microbes-and-Microbiomes.git).

## Results

Our interviews revealed four main themes: (i) previous knowledge, (ii) topic of interest that led to microbial knowledge, (iii) information-seeking processes, and (iv) emotions affecting microbial worldview as indicated in **Table 1**. Additionally, 11 sub-themes, descriptions of each sub-theme, and example quotes are provided in **Table 1**. Sub-theme examples and percent of participants who mentioned each sub-theme example are listed in **Table 2**.

### Previous knowledge about microbes and microbiomes

To answer question RQ1, we analyzed what participants knew about microbes and microbiomes, particularly their features or functions. The data revealed that many participants were aware of the connection of microbes and disease, but many participants were confused about the definition and differences between different types of microbes (i.e. viruses, bacteria, and fungi).

“… I don’t think I know the difference between like bacteria and viruses and microbes and how they all like interact.” (P11)

“I don’t know if they [mushrooms] are microbes…” (P10)

Despite this lack of clarity around definitions, nearly all participants were able to identify a positive function of microbes (93%) or microbiomes (93%), citing familiarity with the existence of “good” and “bad” microbes.

“… I’m aware that they’re not all bad, and I think I’ve heard somewhere that it’s like 80% of microbes or maybe even higher than that are beneficial or completely neutral to the human body.” (P12)

Furthermore, some individuals are unfamiliar with term “microbiome” but can describe what a microbiome is often using word etymology or describing a microbiome without using the term.

“I’m not familiar with [what a microbiome is], but I might assume that… they’re a group or a general environment description for microbes, their atmosphere or their context.” (P5)

Other participants had heard of the term and could provide functions of the microbiome usually associated with the human gut microbiome.

“…it [the human gut microbiome] impacts other systems of your body. So, if you have the right bacteria swirling around in there and your immune system might be better… gut health is actually connected to not having Alzheimer’s … It can potentially impact a lot of your other systems and your ability to stay healthy.” (P4)

Despite some participants not being able to define the terms “microbes” or “microbiomes”, all participants were able to identify at least one positive, negative, or neutral feature and function of both. Most participants (83%) described microbes as “small” or “something you can’t see” and 70% mentioned disease as a negative function. More participants described neutral (100%) or positive (93%) features and functions of microbiomes than negative (53%) features and functions.

### Past information-seeking processes and topics of interest

In response to interview questions related to RQ2, about where and why they learn about microbes and microbiomes, participants describe learning this information through formal education, word of mouth, books, research articles, news media, TV/movies, podcasts, social media, the internet, and advertisements for products. Overall, the concept of microbes was learned through formal education. Alternatively, the concept of microbiomes, specifically the gut microbiome, was learned through social media or advertisements. Most participants stated they learned microbial information from formal education (80%) but either currently receive information from social media or suggested sharing microbial information via social media (56.7%). During an interview, a participant mentioned prebiotics and probiotics; upon further questioning from the interviewer about where they had seen information about this, the participant said:

“Pretty much everywhere now… The health food store that I go to in [a city in the east coast].” (P25)

There were also individuals who displayed source amnesia, an inability to recall where, when or how information has been acquired, which can result in the spread of misinformation [61].

“Definitely personal conversations. It’s hard to pinpoint. It’s honestly hard to distinguish whether I heard the word “microbe” or if it was lumped into conversations about health in general.” (P11)

Participants described topics of interest that led to microbial knowledge. These topics seem to help introduce people to the concepts of microbes and microbiomes indirectly through other interests in fields such as nature, food systems, or health and wellness. Some specific interests included climate change, gardening, composting, farming or agriculture, exercise and fitness, nutrition and gut health, and an interest in natural remedies or holistic medicine.

“I’m also super interested in being healthy… I’m very active. I go to the gym, so naturally that led to, “OK, what should I be eating?”, which led to, “OK, what’s healthy?”, “What’s not?”, which led to deep dives into “OK, like, this is bad for you?” (P21)

“Because I’ve been so interested in this topic, I would say of gardening and learning about like homesteading naturally leads itself to learning more about the natural gut biome…”. (P22)

**Fig 2:**
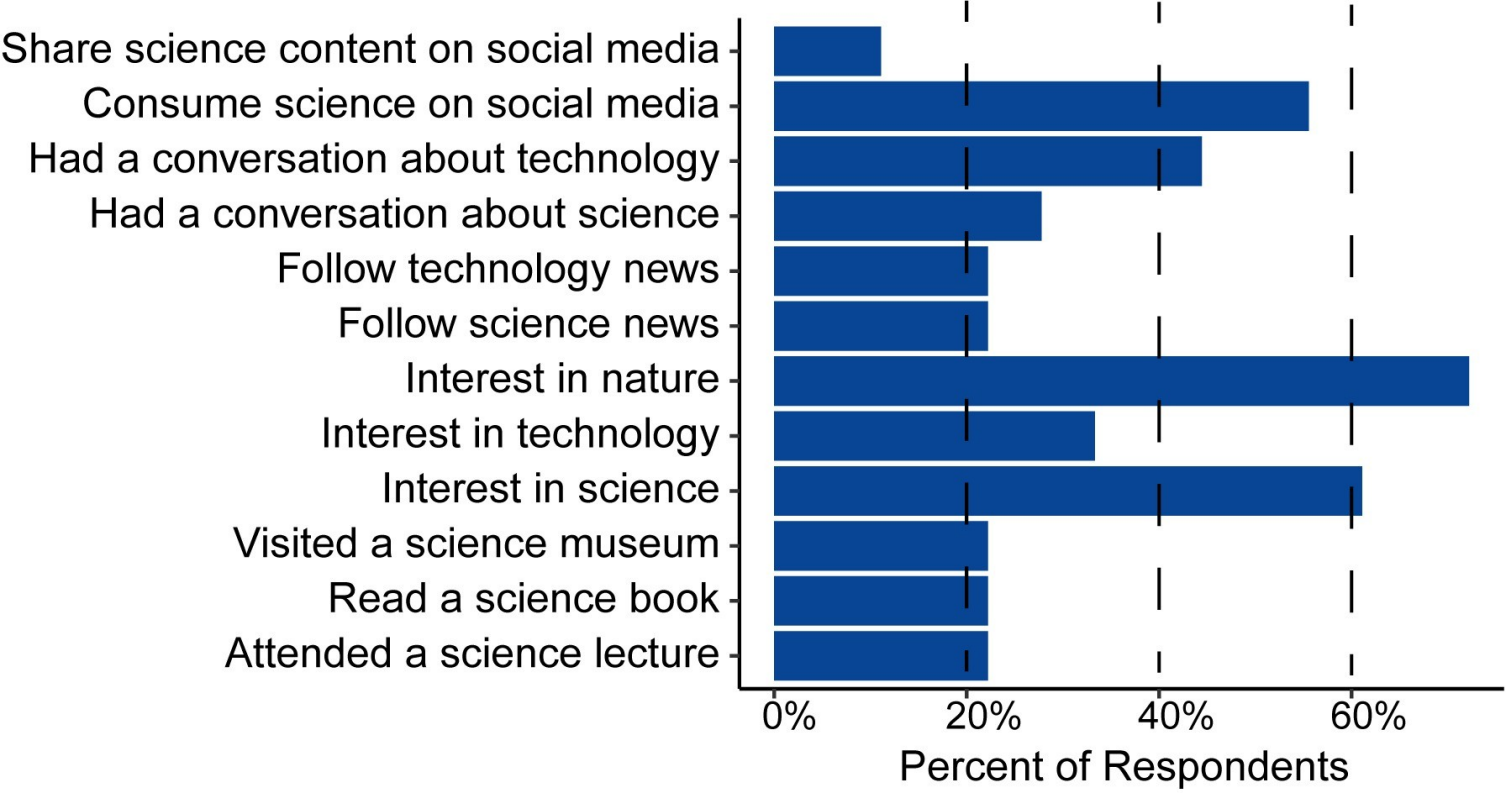
Science curiosity survey question. Bar chart of survey respondents’ (n = 18 participants) science curiosity within the last year. Participants that did not complete the survey (n = 12 participants) are not reflected in this figure.

In the post-interview survey science curiosity question, participants described their science seeking behavior or science interests they participated in within the last year. Over half of survey respondents described having an interest in science (61%), an interest in nature (72%), and consumed science on social media (56%) (**Fig 2**). Collectively, we noticed people with an interest in other fields (n = 19 participants), such as health and wellness, tended to describe more positive functions than negative functions of microbes and microbiomes compared to those who did not mention a special interest (n = 11 participants). Future work using more quantitative methods, such as a survey, should assess whether these trends persist more broadly and are statistically significant. Additionally, people with these interests were more likely to define the term “microbiome” or mention non-human related functions of microbiomes like soil health.

### Desired information about microbes and microbiomes

During interviews, participants shared information they would like to know personally and information they thought others would benefit from (RQ3). Usually, the information a participant thought would be useful for them was described as also being useful for others and related to the perception of others’ knowledge of microbes and microbiomes. The most desired information was general knowledge about microbes and microbiomes such as definitions about these terms. This was often mentioned when describing what others should know about microbes. Many participants (53.3%) also wanted information on disease avoidance and human health benefits. Some participants (16.7%) wanted more microbial information related to climate change solutions or other special interests.

“I would love to learn more… as it relates to climate change and some of the toxic chemicals and products that we have created. And how we could potentially treat those problems with microbes. Also, would love to learn at a high level what we’ve learned as it relates to viruses and immunity and how a better understanding of microbes can help us avoid pandemics in the future.” (P3)

“What are they? What’s their effect on us humans? On animals?… If they are dangerous or not? How to use them in your favor? How to prevent them harming us? Where are they? What is it?… Where do we find them? What’s their lifespan too?” (P5)

More broadly, some participants expressed a desire for themselves or others to understand the ecological roles of microbes (46.7%) and other human-centric benefits (53.3%).

“We should also know they are essential to our existence, because I believe that human existence is dependent on so many factors, and I think microorganisms and microbes are one of the factors. So, I think it will be important for us to also know the role they play in the ecosystem.” (P7)

Those who knew about functions of microbiomes also wanted others to know about the positive role microbes and microbiomes can play.

“I would mainly just like them to know that… it’s a much bigger part of our lives than is readily apparent… I think I would like people to know that they’re not all pathogens. Microbes aren’t just things that cause disease. It’s so much bigger than that.” (P13)

Many participants wanted to learn and share information about how microbes and microbiomes impact human health. Some with an interest in nature emphasized the importance of understanding their role in natural ecological processes or climate change.

### Future information-seeking processes

When describing how they wanted microbe-related messages shared in future science communication efforts (RQ4), participants discussed information sharing channels, communicators, and communication techniques that would be beneficial or should be avoided. Formal education, word of mouth, books, research articles, news media, TV/movies, podcasts, social media, the internet, and advertisements for products were identified by participants as channels to share microbe-related information in the future. Yet regardless of age, there was a greater emphasis on using various social media platforms (Instagram, TikTok, Reddit, etc.) due to perceived reach to a variety of audiences.

“Unfortunately, it [social media] is probably the best place. I hate social media, but I will admit that I am on it. It’s just the quickest access to information when you need it, and social media does a great job of taking information and condensing it into really short videos. That is not great for our attention span; however, if you need a quick answer, it’s good for that.” (P21)

“Reddit. That’s explained like I’m five (years old) in words that I can understand…” (P16)

Participants had a wide array of suggestions as to who they would like to share microbe-related information which included specific individuals they knew, professionals (doctors, scientists, science communicators, or social media influencers), educational institutions (museums, universities, and TED conferences), and governmental institutions (e.g., the Centers for Disease Control and Prevention or the National Institutes of Health). Most commonly, participants wanted the “experts” sharing information about microbes and microbiomes or collaborations between researchers and communicators. The “experts” participants referred to were commonly scientists or others who are “doing the research”, yet some doubted the science communication skills of scientists, suggesting collaborations with individuals with those skills.

“If someone was a microbiologist, if someone was probably a registered dietitian, I’d probably also listen to that… like doctors, like a nurse practitioner or a regular physician.” (P21)

“The information should come from scientists that have the research and things to back it up. I think they’re maybe not the best people to be making those graphics just because they maybe don’t have… the design background. So, I guess it would be someone either from the company or the… marketing department just because I think they have a better idea of how to visually appeal to an audience. I think they should work together.” (P24)

Regardless of the communicator, trust was an important factor described by many participants (46.7%). Some participants were hesitant in recommending government institutions related to how they share information or the public’s trust in these institutions.

“That’s a good question. I feel like if it came from some official source… By official I mean, the CDC or something governmental. Maybe I might be less inclined to click on it, because I might just assume that it was going to be kind of boring. But on the other hand, there needs to be some credibility, because I don’t want to just live in a world where we believe whatever people say on the Internet… I guess there are people who have made careers out of science communication, who have degrees in various science fields, so I guess that’s kind of the group I would look towards.” (P19)

“So, if it’s a government source telling people to do things, I think a lot of people are going to be wary of that just off the bat.” (P22)

Participants also described an array of science communication techniques, most of which have supporting evidence of efficacy [62–67]. Many participants described the effectiveness of “short” video content using “simple” language as an effective means of sharing information.

“I personally just enjoy the video format, and I think a lot of other people do as well. I don’t think I’m just projecting that, maybe I am, but I’m imagining maybe more of a short video. So, think kind of like 5 minutes or less. Think of like Tik-Toks or Instagram reels and very brief discussions that can have powerful visual aids attached to them…. Then you can keep their interest, and they can spend 5 minutes on it, 10 minutes on it.” (P13)

Additionally, some recommended connecting to people’s interests, using humor (6.7%), and having scientists or communicators share their identities outside of their career (16.7%).

“… in a very non-scary, non-sterile way… when I think of people talking about microbiology, I think of people talking jargon and it’s very dry. Maybe there’s a whiteboard behind them and they’re going to show me something with their dry erase marker that I’m probably not going to remember or pay attention to. So… maybe someone who’s dressed in just everyday clothes and they’re like, “Hey. I’d love to tell you something” that would work for me… but if it was told in a way that was humorous, most people are receptive to humor. So, if someone could find a way to say it in a way that’s entertaining like with jokes, that would be great.” (P21)

“I think if you can lay the groundwork of like, “I’m a scientist and I love working in this lab and it’s so great. I love this, and I also love my cat. I love gardening. I love like my grandma and her cookies that she makes me”, I just feel like to me that makes them seem like a real human and they’re more relatable to me…” (P22)

Overall, participants described wanting “experts” that they trust and connect with to be sharing information about microbes and microbiomes. They describe using literature supported science communication techniques and prefer a video format possibly via social media to share this information.

### Emotions affecting microbial worldview

Emotions affect how and when we perceive, seek, and obtain information [24, 25, 41]. Given the role that emotions can play in information processing, questions related to RQ5 help us understand how the publics’ emotions affect their microbial worldview and perceive others. These emotions related to microbial information fell into two main categories: emotions towards self and perceptions of others. Many participants mentioned a variety of emotions throughout the interview. The most common emotions towards self were a lack of self-efficacy (96.7%), apathy (56.7%), curiosity (43.3%), self-efficacy (43.3%), cognitive overload (26.7%), and fear (23.3%) about microbes and microbiomes. Self-efficacy has been defined a person’s perceived competence in their ability to perform a task or achieve a desired outcome, in this case, their ability to understand or explain microbial knowledge, which can affect information management [23, 41, 68–70]. Some participants felt lack of self-efficacy towards some topics but presence of self-efficacy towards other topics. Predominately, individuals experienced a lack of self-efficacy during interviews, with some doubting their own abilities and knowledge of the subject and expressing that they felt ignorant in front of the interviewer. Others had this lack of self-efficacy transform into curiosity about microbes, or they portrayed experiencing cognitive overload.

“I feel like I have an okay grasp on how digestion is affected by them [microbes]. I’d be curious if it is affecting other systems in the body. I am curious about… what they’re doing in the air if they’re doing anything… I guess I’d be curious what advances are being made right now… like fuel or climate-related things… I don’t know if they’re working on like cancer treatments or stuff like that.” (P18)

“One of the hurdles that you have is that you don’t know who’s putting up material. The individuals, or if they represent an organization and many times it’s very contradictory. That one person is telling you to do this, and the other person is telling you, “No, do that”. It’s a juxtaposition between the two of them. They’re clashing, and I have no idea as to who I am going to listen to at this point. Do I listen to one? Do I listen to the other? Do I listen to neither? They [the public] can’t listen to both, because the information is contradictory.” (P27)

Participants’ perceptions of others fell into six categories: disease-focused, ignorant, intimidation, apathy, knowledgeable, and expertise. Most participants (90%) assumed that others knew about as much as or less than they knew about microbes and microbiomes.

“I definitely think they if they’re not like you studying this [the interviewer], they know just as much as me, which is nothing or less than that. Maybe I just think that to make myself feel better, but I do. I feel like people don’t really know what a microbe is, especially since they’re [microbe related products] something we advertise to people…[the term “microbe”] is a buzzword sometimes. I don’t think people know very much about them.” (P9)

Similarly, some participants (56.7%) described feelings of apathy in themselves and would often ascribe it to others as well (50%).

“This probably isn’t a good answer, but I’m kind of apathetic. There’s not like anything I need to know.” (P30)

“My guess [is] the average Joe couldn’t care less about it.” (P26)

Participants (70%) perceived those with a background in science, technology, engineering, or math (STEM) fields or a medical degree as having microbial “expertise”. At the same time, some participants (66.7%) noted that people without a STEM background are knowledgeable about microbes, primarily through news media or experience with disease.

“I think a lot of people don’t know what they are. They’re not scientists… it [the term “microbes”] sounds intimidating and complicated, so I feel like, unless you’re a scientist… maybe if you have a special interest in that kind of research, you probably just know that they are small.” (P24)

“I feel like at this point with COVID, we’ve all had a lot of knowledge or education about cover[ing] your cough, wear[ing] a mask, stay[ing] home when you’re sick. Although a lot of people still aren’t doing those things. So, I guess we could probably use a reminder now and then.” (P19)

Generally, participants thought that others had equivalent or less knowledge than they did about microbes and microbiomes. These perceptions did not apply to those who study microbes and microbiomes. Some describe feeling apathetic, fearful, or overwhelmed with information about microbes and microbiomes while others felt curious or confident about their knowledge especially when it related to other interests that they had. Understanding emotions that underlie the publics’ perceptions of microbes, microbiomes, and others illustrates potential barriers and opportunities for science communication.

## Discussion

With microbial research constantly evolving and an unprecedented wealth of information available, it is overwhelming for both scientists and non-scientists to navigate information-seeking and sharing about microbes and microbiomes. Uncertainty in science is an ever-present reality that challenges science communication efforts [25, 38–41]. Similarly, there is much investigation about how to communicate concepts about microbes and microbiomes [20, 22, 29–32], but many of these studies have focused on a particular infectious disease and have not addressed microbial worldviews holistically. This study investigates public perceptions of microbes and microbiomes through semi-structured interviews and a survey, identifying current microbial knowledge, channels of microbial information, and future directions for science communication. Using the Theory of Motivated Information Management–which connects how people handle uncertainty, emotions, and trust in the efficacy of information providers–as well as Lasswell’s model of communication–which analyzes how messages from particular communicators to particular audiences have certain impacts–as theoretical foundations, we evaluated how individuals’ emotions affect their microbial worldview [41, 48, 49]. We show that stakeholders are considering what, when, how, and who should be sharing microbial information guided by their personal experience.

### Targeting audience pre-existing and desired future knowledge

To generate effective science communication strategies about microbes and microbiomes, it is important to identify audience’s existing knowledge and knowledge gaps (RQ1). Similarly, it is also beneficial to identify what people want to know or think their peers should know about the microbial world (RQ3). Our work notes that while individuals are familiar with the relationship between microbes and disease at a broad level, some participants are also aware of some positive functions of microbes and microbiomes. Creating more awareness of the many industries that microbes already are a part of, such as food, cosmetics, or pharmaceutical industries, may garner greater appreciation and less fear of microbes [9, 14, 35, 42, 71].

Most participants wanted to know general information about microbes and microbiomes including definitions. The data indicate that participants may not know distinct differences between microbes or are unfamiliar with microbe-related terms. Understanding general knowledge about microbes and microbiomes was cited by almost all participants (76.7%) as important for microbial messaging. Lacking this knowledge may lead to confusion about therapeutic interventions or science communication efforts using these terms. For example, other studies have shown low perceived risk and knowledge about antibiotic resistance despite it being among the top ten threats for global health [72–74]. Misconceptions about antibiotics and what types of microbes they are effective against may lead to antibiotic misuse [72, 73]. Clarifying this confusion with targeted interventions may reduce misconceptions and improve health behaviors around issue like antibiotic resistance.

Interestingly, many participants mentioned other interests they had that led them to microbial knowledge. Interests related to human health, specifically nutrition and gut health, and holistic and natural remedies were most common among participants. These interests acted as a catalyst for learning microbial information through leisure activities. These participants were also more aware of functions of microbes and microbiomes typically related to their interests. This corresponded with what participants wanted to know about microbes which generally had a human-centric focus, either as pathogens or how they benefit us. Specifically, people wanted information they could use that could directly impact their lives, which includes disease prevention messaging. Describing how microbes and microbiomes play a role in systems that individuals already have an interest in may be an effective way to share microbial information, and such a strategy could be applied for introducing other scientific topics that have indirect or direct relationships to areas of interest for specific audiences. Deliberate science communication efforts targeting information the public wants to know will aid in crafting meaningful messages which can result in increased scientific engagement.

### Aligning microbial messaging to socio-scientific issues using relevant channels and social networks

When asking individuals where they learned information about microbes and microbiomes (RQ2) and where information should be shared in the future (RQ4), many mentioned school settings. Science education research has identified multiple intervention strategies to improve science literacy in the classroom [7, 8, 42, 66, 71, 75–78]. While it is important for school-aged children to learn microbial information, research and circumstances arise where scientific consensus about microbes changes, and there is still a need to share information with those no longer in school.

When describing instances where participants received knowledge as adults, information was received via social media, internet searches, and word of mouth which can include more misconceptions or misinformation [10, 11, 27, 28, 34, 79, 80]. Social media has contributed to the spread of misinformation, and much research has gone into how to address misinformation on social media [10, 27, 28, 36, 37, 81]. Even though other research and our participants both perceive that social media is not a trustworthy way to receive information [82], our participants report consuming and suggest sharing science information via social media. The reach of social media to more individuals was mentioned as a benefit of social media as a channel. Recommendations include working with content creators that individuals already engage with to broaden the reach of science messaging. These channels are spaces where individuals already received information and can be targeted by science communicators in collaboration with existing content creators to correct misinformation. Engaging with the public and correcting misinformation on social media will require more research to identify best practices and will doubtlessly result in missteps as it may prove difficult to craft messaging suited for a broad audience. Although, choosing to join the conversation provides a chance to improve the ongoing discourse.

Additionally, empowering individuals to correct misinformation within their social groups or social media has been proposed as a strategy and highlights the importance of science literacy and identity [11, 62, 83–87]. Particularly, individuals with interests that led them to microbial information often wanted others to know about the positive functional roles of microbes and microbiomes. They often described sharing what they learned to others through personal interactions. Other participants explained learning about microbes and microbiomes through personal interactions with family or friends with special interests or science education. These relationships may be utilized to share messaging to those within social networks. Future work should examine the role of these interactions in acquiring microbial knowledge compared to other non-social learning methods. Knowledge about where individuals receive information about microbes and microbiomes and their interests can be leveraged for future science communication campaigns by tapping into existing channels of sharing information, topics that individuals will seek out, or social networks.

### Fostering collaboration between microbiologist, social scientists, and communicators

When examining who participants want communicating microbial messages (RQ4), our results highlight the importance of scientists and doctors as a part of the science communication efforts [88–90]. Participants’ perceptions of others were often worse or similar to how participants viewed themselves, which did not apply to how participants viewed those with a STEM background. Many individuals desired information to come from researchers with scientific training. Any reservations about scientists and doctors sharing this information related to the delivery of the microbial information. While participants wanted scientists and doctors to share microbial information, there was a lack of confidence in how effectively those messages would be shared. Scientists were often stereotyped as passionate but boring communicators [91, 92]. To address this, science communication initiatives like “TED talks” have shown success in sharing science stories with non-scientists [65, 93, 94], yet further work needs to be done to communicate research in realistic and entertaining ways. Furthermore, the expertise of those in communication fields are needed to refine science messaging and incorporate best practices. Participants want concise messaging from trusted sources in plain language with exciting visuals. Communicating in this way requires skill, knowledge of your audience, and expertise in evidence-based science communication. This may be done in through integration of science communication into formal scientific training which has been shown to increase science identity and inclusion in science [95–100] as well as collaboration between communicators and scientists. Some scientists’ perceptions of the public as unable to comprehend or uninterested in science provides another barrier to science communication [85, 101–103]. Stepping away from the deficit model of science communication and valuing the expertise of the public would encourage scientists to engage in conversations with the public but also may increase their willingness to correct misinformation [85, 98]. Our data also suggest that scientists should embrace their identities outside of science to increase their relatability and humanity when participating in science communication, which enables connection with others holding similar identities [98, 104–106]. Such boundary spanning may have the added benefit of encouraging others with a shared identity to pursue a career in science [96, 97, 107, 108].

When advising scientists and doctors as communicators, participants typically mentioned the concept of “trust” and “trusted sources”. People tend to trust others they have a personal relationship with but trust different professionals based on their profession [11, 34, 82, 83]. For example, despite a slight decline in confidence in recent years, public trust in scientists and medical scientists remains high while trust in journalists and political leaders is comparatively low [82]. In accordance with this research, some participants advised against sharing science messaging from governmental institutions stating that others may view those sources as more biased [82, 109]. Participants instead recommended sharing information using channels that individuals already engage with, specifically citing social media. Some participants also mentioned specific social media influencers as trusted sources, typically associated with other interests. Benefits of scientists engaging on social media platforms include dispelling misinformation, increasing the visibility of research findings, and professional networking [110–112]. Despite this, many scientists remain reluctant to share science on social media platforms, not knowing where to begin, worrying about contribution to misinformation through unclear language and struggling to incorporate it into the workday [110, 111, 113]. Through collaboration with institution communicators and existing content creators as well as promoting media training for scientists and graduate trainees, scientists can increase their participation in science conversations on social media and through relationships with people in their daily lives.

### Appealing to people’s values and emotions

Perceptions of self and others impact information processing and must be understood when crafting communications (RQ5) [23, 85, 100, 107]. Feeling apathetic, fearful, or overwhelmed with information about microbes and microbiomes can inhibit information processing [24–26, 114, 115]. Additionally, increased levels of intolerance to uncertainty are associated with reduced self-efficacy levels [23, 69, 70]. Addressing negative emotions through targeted messaging can improve understanding and appreciation for science [25, 71, 115–117]. Creating relevant, unified messaging that addresses uncertainty will increase public understanding and self-efficacy [25, 35, 38, 39]. While participants viewed those with STEM trainings as “experts”, they often lacked self-efficacy, which impacts perceptions and health behaviors [23, 35, 42, 107]. Participants often had a lack of self-efficacy, doubting their abilities to obtain or understand microbial knowledge, especially when they did not express an interest that led them to microbial knowledge. Adult learning research indicates, as our participants recommended, that “fun” increases motivation and attention, improving learning outcomes [67, 118]. Connecting microbial information to fields that interest people already can make the “intimidating” task of learning about microbes and microbiomes fun. Future work should examine the benefits of interventions that connect microbial information to participants other interests and subsequent effects on emotions as well as benefits of interventions connecting microbial researchers to people with shared identities via boundary spanning.

### Limitations

According to our data, our participants are highly educated with 80% of our participants having a bachelor’s degree or higher compared to the United States as a whole (37.7%) [119]. These higher education demographics may be elevated since participant recruitment occurred in the vicinity of a college town in a highly educated state. Confidence in scientists has been shown to increase correlated to education level and income, which may have affected participant responses [120]. As mentioned in the methods, our survey response rate (60%; 18/30 participants) limits the conclusion that can be made about demographics-based information. Capturing perspectives of older individuals, individuals from more regions of the United States, individuals without college education, non-white individuals, and more men may identify additional themes not depicted here. Future work should examine how generable these themes are to others across the United States through large scale surveys and assess active messaging to specific demographics groups. Additionally, participants responded to a flyer about microbes and microbiomes which might attract individuals who have some knowledge about them. While this study provides a basis for the publics’ perceptions of microbes and microbiomes, future study design can test proposed interventions to provide relevant microbial science communication to specific stakeholders and communities.

## Conclusion and establishing the groundwork for future research

Together, this work identifies the importance of 1) recognizing the pre-existing and desired future knowledge of audiences (RQ1/RQ3), 2) connecting with larger socio-scientific issues people already care about on social media and through social networks (RQ2/RQ4), 3) collaboration between microbiologists, social scientists, and communicators (RQ4), and 4) connecting with people’s values and emotions (RQ5). Specifically, interests like nutrition and gut health may lead people to microbial knowledge and may be leveraged to share microbial messaging. People may benefit from knowledge about differences between microbes and the ecological functions they have in systems that people value. Future work should examine public knowledge quantitatively to delve deeper into science literacy and its relationship to motivated information seeking. Additionally, our results indicate that individuals want this information communicated on social media and from scientists with whom they share social identities. Research into best practices for communicating microbial topics on social media and careful crafting of messaging should be considered when communicating online as not to contribute to misinformation or the erosion of trust in scientists. Seeing the humanity of scientists promoted trust and better communication outcomes. Information should be unified, clear, and fun to mitigate the distress of scientific uncertainty and other negative emotions while increasing self-efficacy and science literacy. For the most effective communication, scientists should partner with science communication researchers and communicators, implementing tested communication techniques targeted to specific audiences. These findings set the foundation for research to guide future microbial science communication interventions. With this qualitative study, we have identified several themes and topics of interest to audiences, which sets the foundation for quantification, via large landscape surveys. Additionally, future work should examine the complex interplay of social and culture factors that may influence information seeking and processing to develop audience specific messaging. Overall, this work highlights how non-microbial related interest may lead people to microbial knowledge and gives an overview of desired microbial communication topics and techniques. Ultimately, implementation of this work will lead to improved appreciation for and working knowledge of microbes and microbiomes.

## Supporting information

S1 File Final interview facilitation guide. (DOCX)

S2 File Post-interview survey questions. (DOCX)
