# Supplementary material for: Examination of public perceptions of microbes and microbiomes in the United States reveals insights for science communication
Source: PLoS One. 2024 Oct 21;19(10):e0312427. doi: 10.1371/journal.pone.0312427 (PMC11493282; doi:10.1371/journal.pone.0312427)
Supplement: S1 File — (DOCX) [file pone.0312427.s001.docx]

**Facilitation Guide**

**Event Date: Various Dates**

**Arrival Time: 10 mins prior to event**

**Where: Zoom link or in-person depending on participant preference**

**Purpose**

To understand the perception of the public on microbes and microbiomes.

“I am not looking for right or wrong answers. I want to hear about your perspective. What questions do you have for me at this point? Would you mind if I record this, so I can go back and make sure I captured what you said?”

### **Section 1: Introductions (~5 mins)**

**Purpose:** Allow participant to introduce themselves and educational background.

**Facilitator Questions:**

1. Initial question- **What is your profession and educational background?**

### **Section 2: Assessment of Familiarity with Terms (~5 mins)**

**Purpose:** Assess familiarity with microbes (bacteria, fungi, viruses) and the concept of microbiomes (communities of microbes). To identify channels of communication or specific science communicators. To understand perception of others with regards to microbes and microbiomes.

**Facilitator Questions:**

1. Initial question- **What comes to mind when you think of microbes?** **Microbiomes?**
2. **How would you define a microbe?**
   1. What have you heard about microbes? Where did you learn this information?
3. **How would you define a microbiome?**
   1. What have you heard about microbiomes? Where did you learn this information?
   2. If “I haven’t about microbiomes” --> If you had to guess, what do you think that might mean?
   3. *If they discuss interest in nutrition or alternative medicine, ask about their perspective on these fields.*
4. **What do other people think of microbes? Microbiomes?**

### **Section 3: Abundance and Roles of Microbes and Microbiomes (~5-10 mins)**

**Purpose:** Assess familiarity with the abundance and roles microbes (bacteria, fungi, viruses) and microbiomes (communities of microbes)

**Facilitator Questions:**

1. **Where are microbes found?**
2. **Why do people study microbes and microbiomes?**
3. **Describe an example of how microbes can be used in different industries.**

Additional questions rephrased: How can microbes be used to help people? Animals? The planet?

1. **I am going to ask you a few statements, please tell me if you agree or disagree with the statement and why.**
   1. All microbes are dangerous
   2. Soil is sterile
   3. Microbes promote health
   4. Microbes help plants grow better
   5. I interact with microbes every day

### **Section 4: Future Science Communication (~10 mins)**

**Purpose:** Determining what people would like to know more about, who should deliver that information, and how that could be delivered. To identify channels of communication or specific science communicators. To understand perception of others with regards to microbes and microbiomes.

**Facilitator Questions:**

1. **What would you like to know about microbes or microbiomes? How would you go about getting that information?**
   1. How would you want to receive this information? What would you say is an ideal way to receive this information?
2. **What do you think other people should know about microbes/microbiomes?**
   1. How would we engage with people about microbes and microbes?
3. **Who should be sharing this information about microbes? Microbiomes?**
