# Supplementary material for: Examination of public perceptions of microbes and microbiomes in the United States reveals insights for science communication
Source: PLoS One. 2024 Oct 21;19(10):e0312427. doi: 10.1371/journal.pone.0312427 (PMC11493282; doi:10.1371/journal.pone.0312427)
Supplement: S2 File — (DOCX) [file pone.0312427.s002.docx]

Thank you for participating in the interviews about your perception of microbes. We would like to understand the demographics of our interview participant pool. We would like you to answer the following demographic questions, which should take ~5 minutes. There are no direct benefits to you, but understanding the demographics of our interview participants will help us better understand how different members of the public understand microbes. There are no known risks included with answering these questions. You will not receive any compensation for answering these questions. You can choose to not answer any question.

All information gathered in this study will be kept as confidential as possible. Your privacy is very important to us and the researchers will take every measure to protect it. Your information may be given out if required by law; however, the researchers will do their best to make sure that any information that is released will not identify you. No reference will be made in written or oral materials that could link you to this study. For this study, we will assign a code to your data so that the only place your name will appear in our records is on the consent and in our data spreadsheet which links you to your code. Only the research team will have access to the link between you, your code, and your data. All records will be stored in a password-protected drive at CSU for three years after completion of the study. After the storage time, the information gathered will be destroyed.  We may be asked to share the research files with the sponsor or the CSU Institutional Review Board ethics committee for auditing purposes.

Your demographic information will not be shared for any future study.

**WHAT IF I HAVE QUESTIONS?**

 For questions or concerns about the study, you may contact **Nicole Kelp** at [nicole.kelp@colostate.edu](mailto:nicole.kelp@colostate.edu)

For questions regarding the rights of research subjects, any complaints or comments regarding the   manner in which the study is being conducted, contact the CSU Institutional Review Board at:  [CSU_IRB@colostate.edu](mailto:CSU_IRB@colostate.edu); 970-491-1553.

**Do you consent to proceed to the demographic survey?" (answers yes/no)**

**Question 1:**

Gender. What is your gender?

Male

Female

Non-binary

Other

**Question 2:**

Age. What is your age?

18-24

25-34

35-44

45-54

55-64

65-74

75 or older

**Question 3:**

Ethnicity. How would you describe yourself? Please select all that apply.

White

Black or African American

American Indian or Alaskan Native

Asian

Native Hawaiian or Pacific Islander

Latino, Hispanic, or Spanish origin

Middle Eastern or North African

Other

**Question 4:**

Location. What city and state do you live in? (ex. Fort Collins, CO)

**Question 5:**

Education. What is the highest degree or level of school you have completed?

Less than a high school diploma

High school degree or equivalent (e.g. GED)

Some college, no degree

Associate degree (e.g. AA, AS)

Bachelor’s degree (e.g. BA, BS)

Master’s degree (e.g. MA, MS, MEd)

Doctorate or professional degree (e.g. MD, DDS, PhD)

**Question 6:**

Science Curiosity. Which of the following science related activities have you done in the past year? Select all that apply.

Attended a science lecture this year

Read a science book this year

Interest in science

Follow technology news

Visited a science museum this year

Interest in technology

Interest in nature

Conversation about science

Conversation about technology

Follow science news

Share science content on social media (YouTube, Instagram, Twitter, etc.)

Consume science content on social media (YouTube, Instagram, Twitter, etc.)
